# Urinary Sodium and Potassium Excretion and Carotid Atherosclerosis in Chinese Men and Women

**DOI:** 10.3390/nu8100612

**Published:** 2016-10-01

**Authors:** Xiao-Wei Dai, Cheng Wang, Ying Xu, Ke Guan, Yi-Xiang Su, Yu-Ming Chen

**Affiliations:** 1 Guangdong Provincial Key Laboratory of Food, Nutrition and Health, School of Public Health, Sun Yat-Sen University, Guangzhou 510080, China; dxwpc2004@126.com (X.-W.D.); wangch28@mail2.sysu.edu.cn (C.W.); xuying_qiang@163.com (Y.X.); guanke@163.com (K.G.); 2 Nanshan Maternal and Child Health Hospital of Shenzhen, Shenzhen 518067, China; 3 Department of Nutrition, School of Public Health, Sun Yat-Sen University, Guangzhou 510080, China; 4 Department of Medical Statistics & Epidemiology, School of Public Health, Sun Yat-Sen University, Guangzhou 510080, China

**Keywords:** sodium, potassium, carotid atherosclerosis, Chinese adults

## Abstract

Limited studies have examined the association between sodium (Na) and potassium (K) levels and the risk of atherosclerosis. This study examined whether higher Na and Na/K levels and low K levels were independent risk factors for atherosclerosis. This community-based cross-sectional study included 3290 subjects (1067 men and 2223 women) 40 to 75 years of age in Guangzhou, China, between 2011 and 2013. Urinary excretion of Na and K were measured from the first morning void, and creatinine-adjusted values were used. The intima-media thickness (IMT) of the carotid common artery and the carotid bifurcation was measured with high-resolution B-mode ultrasonography. Dietary K and Na intake and other covariates were obtained by face-to-face interviews. A significant positive association was seen between urinary Na excretion and carotid atherosclerosis after adjustment for age, sex, and other lifestyle covariates. The odds ratios (OR) and 95% confidence interval (CI) of the highest (vs. lowest) quartile of urinary Na were 1.32 (1.04–1.66) for carotid plaques, 1.48 (1.18–1.87) for increased common carotid artery IMT, and 1.55 (1.23–1.96) for increased carotid bifurcation IMT (all *p*-trend < 0.01). A similar positive association was observed between urinary Na/K levels and carotid plaque and increased IMT, and between dietary Na intake and increased bifurcation IMT. Regarding potassium data, we only found a significantly lower presence of carotid plaque (OR 0.72, 95% CI 0.57–0.91) for quartile 2 (vs. 1) of urinary K. Our findings suggest that higher levels of urinary excretion Na and Na/K are significantly associated with greater presence of carotid atherosclerosis in Chinese adults.

## 1. Introduction

A direct relationship between high levels of dietary sodium intake and the prevalence of hypertension has been repeatedly demonstrated across populations. The results of a recent meta-analysis of 36 randomized controlled trials documented a consistent effect of sodium on blood pressure in subjects with hypertension and in normotensive subjects [1]. Because hypertension contributes to the risk of cardiovascular disease (CVD), a reduction in sodium intake could be associated with a decreased risk of CVD. Some prospective studies have shown similar results [2,3,4,5], but many others failed to identify any significant association between dietary salt intake and cardiovascular events [6,7]. Moreover, a few studies showed a negative association between dietary sodium intake and mortality from all causes in the setting of type 2 diabetes [8] or a J-shaped association between urinary excretion of sodium and CVD events in subjects with a high risk of CVD [9].

The measurement error in the estimation of an individual’s usual sodium intake may lead to attenuation of the associations between sodium intake and CVD risk toward the null, especially in studies in which sodium intake is assessed by means of a food frequency questionnaire (FFQ) [6]. In addition, a narrow range of sodium intake [7], greater sodium sensitivity in obese persons [4], and heterogeneity of the CVD risk in study samples [8] may also modify the association between sodium intake and CVD. Nonetheless, most of these studies were conducted in Western countries. Because of the high intake of sodium in China [10,11] and the potential for ethnic variations, it is important to determine whether sodium intake is associated with the risk of CVD in the Chinese population.

A high intake of fresh fruits and vegetables is always accompanied by high intake of potassium. Therefore, an increase in dietary potassium intake is expected to exert a protective effect against CVD. The favorable effects of a high potassium intake for the prevention of stroke have been shown in a meta-analysis of 11 prospective studies [12]; lesser benefits for coronary heart disease (CHD) and total CVD were also shown [12].

Atherosclerosis is an early stage of CHD and stroke, but little is known about the association of potassium intake with atherosclerosis. This study aimed to assess the hypothesis that a high intake of sodium, a high sodium/potassium ratio, and low intake of potassium, using urinary excretion to estimate sodium and potassium intake, were associated with a greater presence of atherosclerosis in middle-aged and older Chinese adults.

## 2. Methods

### 2.1. Study Participants

This community-based cross-sectional study was based on the Guangzhou Nutrition and Health Study (GNHS), a cohort study designed to assess the determinants of cardiometabolic outcomes and osteoporosis. The study included 3169 participants 40 to 75 years of age from urban Guangzhou, China, recruited between 2008 and 2010 via subject referral and community advertisement. Participants with hospital-confirmed diabetes mellitus (DM), CVD, renal failure, chronic kidney disease (CKD) or cancer were excluded. DM was defined as either fasting glucose (FG) ≥7.0 mmol/L, or use of insulin or anti-diabetic medication use [13]. Patients for whom CVD were identified by the International Classification of Diseases (ICD-10 codes I00-I78) were also excluded. Between April 2011 and May 2013, 2510 participants (79.2%) completed the first follow-up survey at a mean (SD) interval of 3.2 (0.5) years of follow-up. To complement the subjects lost during follow-up, we recruited 869 additional participants from March 2013 to October 2013 according to the baseline criteria. Cross-sectional data were used for this study from the subjects followed-up, and the new subjects collected during 2011–2013. Written informed consent was obtained from all participants at initial enrollment and at follow-up, and the study was approved by the Ethics Committee at SunYat-sen University.

### 2.2. Data Collection

The eligible subjects were invited to the School of Public Health of Sun Yat-sen University. Face-to-face interviews were conducted by trained medical staff using a structured questionnaire to collect data on dietary nutrient intake, socio-demographic variables, general risk factors for CVD such as smoking habits and alcohol use, education level, monthly family income, physical activity, history of disease, and menopausal status.

Dietary sodium and potassium intake from foods during the past year was assessed with an interviewer-administered 79-item FFQ and calculated on the basis of the Chinese Food Composition Table 2002 [14]. The relative validity and reproducibility of the FFQ were confirmed in a previous study [15]. The energy-adjusted correlation coefficients were 0.64 and 0.57 for fruit and vegetable intake between two FFQs and 0.56 and 0.37 when the FFQ was compared with six 3-day dietary records, respectively [15]. The participants were asked to report the usual frequency of consumption of each food item (“never”, “per year”, “per month”, “per week”, or “per day”) and the mean portion size, which was estimated using color photographs. All nutrients were adjusted for total energy intake using the residual method [16].

Body weight and height were measured while the subjects were wearing light clothing and no shoes, and the body mass index (kg/m^2^) was calculated. The waist and hip circumferences were measured at the midline between the costal margin and the iliac crest and at the point of maximum girth around the buttocks, respectively. The blood pressure was measured on the left arm with a digital sphygmomanometer (HEM-7011, OMRON Corp., Osaka, Japan) after the participants sat still for at least 10 min. Each measurement was taken twice, and the average was calculated. Subjects who smoked at least one cigarette per day or drank alcohol once a week for at least 6 months were defined as smokers or drinkers. Daily physical activity was estimated as previously described [17], and the metabolic equivalent intensity (excluding time spent sleeping and sitting) was calculated.

### 2.3. Laboratory Assay

A first morning fasting urine sample was obtained from 3290 participants to estimate their salt intake. The sodium and potassium concentrations in each urine specimen were determined by the ion selective electrode method (Hitachi Ltd., Tokyo, Japan), and the creatinine concentration was determined by enzymatic colorimetric assay (Sekisui Chemical Co., Ltd., Tokyo, Japan) with a Hitachi 7180 automatic analyzer. The coefficients of variation were assessed by repeated analysis of pooled samples. The within-run imprecisions of urinary sodium, potassium, and creatinine excretion were 1.45%, 1.63%, and 1.99%, respectively; and the between-runs imprecisions were 7.04%, 7.24% and 7.23%, respectively. The ratios of urinary sodium/creatinine (Na/Cr), potassium/creatinine (K/Cr), and sodium/potassium (Na/K) were calculated for further analysis.

Venous blood samples were drawn after a 12-h overnight fast for measurement of fasting glucose and lipid concentrations and stored at −80 °C until analysis. Serum levels of fasting glucose, total cholesterol, triglycerides, high-density lipoprotein cholesterol (HDL-c), and low-density lipoprotein cholesterol (LDL-c), and uric acid were measured with commercial kits (Roche Diagnostics, Indianapolis, IN, USA) with an Olympus AU400 automatic analyzer (Olympus Corporation, Tokyo, Japan). The coefficients of variation for the measurements were 4.94% for glucose, 4.55% for total cholesterol, 5.74% for triglycerides, 5.56% for HDLc, 3.36% for LDLc, and 4.67% for serum uric acid.

### 2.4. Measurement of the Carotid Intima-Media Thickness

A total of 2919 subjects underwent measurement of their carotid intima-media thickness (IMT). The carotid IMT was measured bilaterally at the far walls of the common carotid artery (CCA; the 1-cm segment proximal to the bifurcation) and at the carotid bifurcation (the 1-cm segment proximal to the flow divider), using a high-resolution, 6.0- to 11.0-MHz linear-array transducer (SSI-5500, Sonoscape Corp., Shenzhen, China) with the subject in the supine position after resting for 10 min. All B-mode images were recorded during the diastolic phase and read automatically by two professionals. Plaques (defined as focal thickening 50% greater than the surrounding wall thickness) [18] were avoided in the measurement of the IMT. The average measurement of the two sides was used in the analyses. Intrapatient reproducibility was assessed by replicating measurements for 21 randomly selected subjects using a different image from the same day; the site-specific reliability coefficients were 0.94 for the CCA IMT, and 0.92 for the carotid bifurcation IMT, respectively.

### 2.5. Statistical Analysis

All statistics were analyzed with SPSS 16.0 for Windows (SPSS Inc., Chicago, IL, USA), and two-sided *p* values of less than 0.05 were considered to be statistically significant. The data were presented as means (and SD) for continuous variables or proportions (%) for categorical variables. The participants were categorized into quartiles by urinary values of Na/Cr, K/Cr, or Na/K and by their energy-adjusted dietary sodium and potassium intake.

The patients’ characteristics were compared with a chi-square test or analysis of variance among the quartiles of Na/Cr and K/Cr. Multivariate logistic regression models were applied to estimate the odds ratios (95% confidence interval) for the prevalence of carotid IMT thickening at the CCA and at the carotid bifurcation (defined as an IMT of 1 mm or greater on any side) [19] and carotid artery plaque (defined as a focal thickening of 50% greater than the surrounding wall thickness on any side) [18] by comparing the quartiles of urinary Na/Cr, K/Cr, and Na/K and dietary sodium and potassium intake. We constructed the following models sequentially: (1) adjusted for demographics: age and sex (Model 1); (2) further adjusted for lifestyle risk factors: education level (≤6, 7 to 12, and >12 years), monthly income (<1500, 1500 to 3000, and >3000 yuan), smoking (yes/no), alcohol consumption (yes/no), physical activity (metabolic equivalent, excluding sleeping and sitting; hours per week), and dietary factors including total energy (kcal/day), fiber, and saturated fat (g/day) (Model 2); and (3) further adjusted for cardiovascular risk factors: waist circumference, hypertension status (yes/no), antihypertensive medication (yes/no), fasting serum lipids including total cholesterol, triglycerides, HDL-c, and LDL-c, fasting serum glucose and serum uric acid (Model 3). The *p-*values for linear trends across increasing quartiles were determined by assuming the median values of quartiles as continuous variables. All independent variables were entered into the models.

We assessed potential effect modification by sex (male/female), body mass index (<25 or ≥25 kg/m^2^), and hypertension status (yes/no) by including interaction terms between urinary sodium excretion and these variables in the fully adjusted model. For multivariate analysis in women, we further adjusted for menopausal status (yes/no) and passive smoking and excluded adjustment for smoking because only 10 subjects were smokers.

## 3. Results

The 3290 participants in the analyses included 1067 men and 2223 women. The mean (±SD) age of the men was 62.1 ± 6.7 years, and that of the women was 59.4 ± 5.5 years. The participants’ demographic and clinical characteristics, grouped by quartiles of urinary Na/Cr, are presented in Table 1. The participants in the higher quartiles of urinary Na/Cr were significantly older, more likely to be female, and less educated. Moreover, there was a significant increasing trend in the mean values of potassium excretion, waist circumference, body mass index, and systolic blood pressure and in the prevalence of obesity and of carotid atherosclerosis as defined as wall thickening by increasing the Na/Cr ratio. However, the level of serum uric acid was decreased across the quartiles of Na/Cr ratio (Table 1). A higher K/Cr ratio was associated with lower diastolic blood pressure values and a lower prevalence of carotid plaque (Table S1).

In the logistic regression of Model 2, a positive association was observed between urinary Na/Cr and the incidence of carotid atherosclerosis. Compared with the extreme quartiles of urinary Na/Cr, the odds ratios (OR) and 95% confidence interval (CI) was 1.32(1.04–1.66) (*p*-trend = 0.009) for carotid plaque, 1.48(1.18–1.87; *p*-trend = 0.001) for an increase in the CCA-IMT, and 1.55 (1.23–1.96) (*p*-trend = 0.001) for an increase in the carotid bifurcation IMT, respectively. For dietary Na intake, we just found a significant association with increased BIF IMT, the OR (95% CI) was 1.31 (1.04–1.64) (*p*-trend = 0.022) when comparing the extreme quartiles, but not with carotid plaque or CCA IMT. A similar positive association was also observed between the urinary Na/K and the occurrence of carotid plaques. Additional adjustment for cardiometabolic factors did not alter the substantial associations between the Na/Cr ratio and an increase in the IMT at either the CCA or the carotid bifurcation. Regarding potassium of urinary excretion and dietary intake, we found a “J-shaped” association between potassium levels estimated from urine excretion with the presence of carotid plaque (OR 0.72, 95% CI 0.57–0.91 for quartile 2 (vs. 1)). However, no significant association was observed between urinary potassium and the presence of increased IMT, and for the dietary potassium (all *p*-values > 0.05) (Table 2 and Table 3).

To figure out whether the use of diuretics influenced the associations between urinary Na or K and carotid IMT, we conducted analyses by excluding those used antihypertension in Supplementary Materials Tables S2 and S3. The results showed that urinary Na excretion was still significantly associated with increased CCA IMT (*p*-trend = 0.027) and BIF IMT (*p*-trend = 0.006) in Model 2, whereas not significantly associated with the prevalence of carotid plaque (*p*-trend = 0.287). We did not find any statistical associations of urinary K excretion and carotid IMT as before (See Supplementary Materials Table S3).

In a stratified analysis, the association of urinary Na/Cr with the CCA-IMT was more significant in the normal-weight subjects than in overweight or obese subjects (*p*-interaction = 0.006). No significant differences were found in the associations between Na/Cr and IMT between the subgroups by gender and hypertension status (all *p*-interaction > 0.05) (Table 4).

## 4. Discussion

In this community-based cross-sectional study of the middle-aged and older Chinese population, we found a significant positive association between urinary excretion of sodium and the presence of carotid atherosclerosis independent of demographic and socioeconomic factors, smoking and alcohol drinking, and cardio metabolic factors. However, no significant associations were observed between urinary excretion of potassium or dietary potassium intake and carotid atherosclerosis.

Our study found that increased excretion of urinary sodium was associated with a greater incidence of carotid wall thickening. Similar results were observed in several observational studies. A cross-sectional study found that 24-h urinary sodium excretion or urinary Na/Cr ratios were positively associated with carotid IMT in normotensive overweight and obese adults [20]. A recent meta-analysis of cohort studies indicated a 24% greater risk of stroke (95% CI, 8% to 43%) and a 32% greater risk of fatal CHD (95% CI, 13% to 53%) for those with higher sodium intake [1]. A previous meta-analysis had yielded similar results [21]. The benefits of lower sodium intake for atherosclerosis and consequent CVD events were shown to be strengthened in populations or individuals with a high sodium intake, such as those in China [10,11]. Moreover, a meta-analysis of randomized clinical trials showed that a reduction in sodium of 2.0 to 2.3 g/day lowered the risk of CVD (relative risk, 0.80; 95% CI, 0.64 to 0.99) in 3225 participants who were followed-up for seven months to 11.5 years [22]. Overall, our findings and those of previous studies consistently highlight the importance of sodium reduction in the prevention of atherosclerosis and CVD.

In addition, some studies have indicated that reduced sodium intake was especially efficacious for decreasing the risk of CVD in overweight individuals in the United States [4] or Finland [3]. However, in this study, we found that the association between Na/Cr and CCA-IMT tended to be more significant in subjects with a normal weight (body mass index less than 25 kg/m^2^). It is unclear whether different ethnic populations vary in their susceptibility to higher sodium intake. Further studies are needed to clarify this issue.

A high intake of dietary sodium has been shown to have a direct relationship with essential hypertension [1]. A high sodium intake may increase the risk of atherosclerosis partly through hypertension. We found, however, that the association between high levels of urinary excretion of sodium and atherosclerosis was independent of blood pressure and was not modified by hypertension status. Heavy consumption of sodium may have other detrimental effects that contribute to atherosclerosis. Emerging evidence suggests that increased intake of dietary salt in healthy subjects induced rapid expansion of CD_14_++CD_16_+ monocytes (a major player in inflammation) and platelet activation, in addition to monocyte pro-inflammatory activation [23]. The adverse effects of a high sodium intake on the inflammation marker tumor necrosis factor-α were also observed in Sprague Dawley rats with chronic renal failure [24]. An in vitro study demonstrated a direct effect of plasma sodium concentration on endothelial stiffness and a reduction in nitric oxide release from the vascular endothelium [25]. In addition, oxidative stress was increased as a consequence of an increase in dietary salt intake in normal rats [26].

Regarding potassium data, significantly lower presence of carotid plaque was found with the moderate urinary K excretion. Previous studies showed inconsistent results for CVDs. A meta-analysis of 11 prospective studies [12] demonstrated that an increase in potassium intake of 1.64 g/day (42.1 mmol/day) was associated with a 21% decline in the risk of stroke (95% CI, 10% to 32%) and a trend toward risk reduction in CHD (0.92; 95% CI, 0.81 to 1.04) and CVD (0.85; 95% CI, 0.62 to 1.16). However, seven of the 11 studies included did not show a significant association with the risk of stroke, and some did so only for certain population subsets, such as women [5] or subjects with hypertension [27]. In a meta-analysis of 31 randomized controlled trials, supplementation with potassium decreased systolic blood pressure (−3.11 mm Hg; −1.91 to −4.31 mm Hg) and diastolic blood pressure (−1.97 mm Hg; −0.52 to −3.42 mm Hg) by a small but significant amount [28]. It is suggested that the protective effects of potassium intake against the risk of stroke may be attributed mostly to its blood pressure-lowering effect. High blood pressure is responsible for 62% of strokes and 49% of CHD [29]. Because blood pressure was more closely related to stroke than other CVD events, the moderate favorable effects of high potassium intake on blood pressure may translate to a reduction in the risk of stroke, but not in the risk of other CVD events or atherosclerosis. More studies are needed to finally address the potential effects of potassium.

To our best knowledge, this work was the first one to assess the associations between urinary sodium and potassium excretion and increased carotid IMT, a marker for an early stage of stroke and other CVD, in the Chinese with a relatively large sample. However, our study also has several limitations. First, due to the nature of a cross-sectional study, we were unable to determine any causal relationship between urine excretion of sodium and carotid IMT. Studies have suggested that exposure to sodium is relatively constant over time at a population level estimated either by 24-h urine sodium excretion [30] or by 24-h dietary recall [31]. Second, 24-h urinary sodium excretion is the best way to measure an individual’s sodium intake. Previous studies have suggested that the estimation of urinary sodium excretion from overnight urine could be an acceptable alternative to that from 24-h urine with a good correlation coefficient (*r* = 0.73; *p* < 0.001) [32]. Nonetheless, overnight collections have been shown to underestimate 24-h values [33]. Therefore, in the present study, the positive associations between urinary sodium excretion and carotid atherosclerosis might be underestimated. Third, we also used a 79-item food frequency questionnaire (FFQ) to assess the daily intakes of sodium and potassium, and null or moderate associations were found with carotid atherosclerosis, which may be partly due to the large random errors of dietary assessments by the FFQ method. Finally, although we adjusted for major sociodemographic characteristics, lifestyle and cardio metabolic factors, residual confounders, such as unknown or unmeasured factors (e.g., estimated glomerular filtration rate) cannot be completely ruled out, which might mask or attenuate the true associations.

## 5. Conclusions

In summary, our study showed that high consumption of sodium as estimated by excretion from fasting morning urine was associated with an increased presence of carotid atherosclerosis in Chinese adults. However, inconsistent results are generated for potassium intake, as estimated by urinary excretion or by food frequency questionnaire, and more solid studies are needed to clarify it effects on the risk of atherosclerosis in this population.

## Figures and Tables

**Table 1:** Characteristics of participants by quartiles (Q) of urinary Na/Cr (*n* = 3290).

	Q1 Mean ± SD /*n* (%)	Q2 Mean ± SD /*n* (%)	Q3 Mean ± SD /*n* (%)	Q4 Mean ± SD /*n* (%)	*p*-Trend ^a^
Urinary Na/Cr	7.7 ± 2.0	12.9 ± 1.4	18.4 ± 2.0	32.0 ± 17.0	
Age, year	59.3 ± 5.9	59.9 ± 5.7	60.7 ± 6.0	61.4 ± 6.3	<0.001
Male, *n* (%)	373 (45.4)	261 (31.7)	248 (30.1)	185 (22.5)	<0.001
Waist circumference, cm	83.7 ± 8.6	84.6 ± 8.7	85.4 ± 8.6	86.2 ± 9.1	<0.001
Body mass index, kg/m^2^	23.2 ± 3.0	23.5 ± 3.0	23.6 ± 3.1	24.1 ± 3.5	<0.001
Overweight, *n* (%)	210 (25.5)	230 (27.9)	245 (29.8)	301 (36.6)	<0.001
Education >12 years, *n* (%) ^b^	211 (25.7)	221 (26.9)	230 (27.9)	175 (21.3)	<0.001
Monthly income, *n* (%)					0.292
<1500, yuan	59 (7.2)	47 (5.7)	54 (6.6)	64 (7.8)	
1500–3000, yuan	416 (50.6)	385 (46.8)	397 (48.2)	401 (48.8)	
>3000, yuan	345 (42.0)	391 (47.5)	370 (45.0)	355 (43.2)	
Current smoker, *n* (%)	123 (15.0)	89 (10.8)	62 (7.5)	44 (5.4)	<0.001
Alcohol drinker, *n* (%)	67 (8.2)	68 (8.3)	64 (7.8)	59 (7.2)	0.844
Physical activity, MET/day ^c^	24.9 ± 6.4	25.3 ± 7.2	25.0 ± 6.6	24.7 ± 6.6	0.415
Urinary Cr, mmol/L	10.3 ± 5.2	7.1 ± 3.2	5.6 ± 2.4	3.8 ± 1.6	<0.001
Urinary K/Cr	3.0 ± 1.3	3.6 ± 1.7	4.1 ± 1.7	5.4 ± 5.0	<0.001
Urinary Na/K	2.9 ± 1.4	4.1 ± 1.6	5.2 ± 2.0	6.9 ± 2.9	<0.001
Dietary Na intake, mg/day	833 ± 394	864 ± 507	825 ± 412	828 ± 395	0.411
Dietary K intake, g/day	1.98 ± 0.69	2.03 ± 0.85	1.98 ± 0.69	2.01 ± 0.74	0.758
SBP, mm Hg	123.9 ± 17.7	124.1 ± 17.4	124.8 ± 17.9	128.0 ± 18.5	<0.001
DBP, mm Hg	75.4 ± 10.5	75.3 ± 9.7	74.9 ± 10.2	76.3 ± 10.7	0.139
TC, mmol/L	5.47 ± 0.99	5.61 ± 1.04	5.61 ± 1.05	5.58 ± 1.09	0.052
TG, mmol/L	1.51 ± 1.22	1.55 ± 1.34	1.54 ± 1.04	1.55 ± 1.32	0.505
HDLc, mmol/L	1.43 ± 0.42	1.43 ± 0.39	1.44 ± 0.41	1.44 ± 0.41	0.482
LDLc, mmol/L	3.50 ± 0.87	3.62 ± 0.90	3.60 ± 0.88	3.57 ± 0.97	0.124
Fasting glucose, mmol/L	4.92 ± 0.90	5.03 ± 1.24	5.05 ± 1.24	5.14 ± 1.41	<0.001
Serum uric acid, µmol/L	363 ± 89	349 ± 83	356 ± 87	343 ± 82	<0.001
Carotid IMT					
CCA, mm	0.908 ± 0.114	0.921 ± 0.115	0.924 ± 0.118	0.936 ± 0.125	<0.001
BIF, mm	0.944 ± 0.121	0.958 ± 0.113	0.959 ± 0.119	0.974 ± 0.148	<0.001
Plaque, *n* (%)	245 (29.8)	239(29.0)	253 (30.7)	294 (35.8)	0.013

Abbreviations: BIF, carotid bifurcation; CCA, common carotid artery; Cr, creatinine; DBP, diastolic blood pressure; HDLc, high density lipoprotein cholesterol; IMT, intima-media thickness; K, potassium; LDLc, low density lipoprotein cholesterol; Na, sodium; SBP, systolic blood pressure; TC, total cholesterol; TG, triacylglycerol; ^a^ Linear trend across increasing quartiles was tested by assuming median values of quartiles as continuous variables; ^b^ High education level was defined as at least 12 years of education; ^c^ Physical activity included occupational, leisure-time, and household chores, presented as metabolic equivalent (MET) hours per day (excluding sleeping and sitting time).

**Table 2:** Odds ratios and 95% CI for the prevalence of carotid plaque by quartiles (Q) of sodium (Na) and potassium (K).

	Odds Ratios (95% CI) for Carotid Plaque				
	Q1	Q2	Q3	Q4	*p*-Trend ^a^
**Urinary Na/Cr**					
Median	7.9	12.9	18.3	28.3	
Cases/*n*	245/711	239/729	253/696	294/723	
Model 1	1.00	0.94 (0.75–1.19)	1.07 (0.85–1.35)	1.28 (1.02–1.62)	0.018
Model 2	1.00	0.96 (0.76–1.21)	1.12 (0.88–1.41)	1.32 (1.04–1.66)	0.009
Model 3	1.00	0.96 (0.76–1.22)	1.10 (0.86–1.40)	1.26 (0.99–1.60)	0.032
**Urinary K/Cr**					
Median	2.2	3.1	4.1	6.0	
Cases/*n*	290/732	226/723	252/698	263/706	
Model 1	1.00	0.72 (0.57–0.91)	0.95 (0.76–1.21)	0.99 (0.78–1.26)	0.513
Model 2	1.00	0.72 (0.57–0.91)	0.98 (0.77–1.23)	1.01 (0.80–1.29)	0.387
Model 3	1.00	0.73 (0.58–0.93)	0.95 (0.75–1.22)	1.04 (0.82–1.34)	0.324
**Urinary Na/K**					
Median	2.3	3.6	5.0	7.5	
Cases/*n*	221/701	252/712	267/725	291/721	
Model 1	1.00	1.11 (0.88–1.39)	1.10 (0.87–1.38)	1.33 (1.06–1.68)	0.019
Model 2	1.00	1.07 (0.85–1.35)	1.08 (0.86–1.36)	1.32 (1.05–1.66)	0.023
Model 3	1.00	1.04 (0.82–1.32)	0.99 (0.78–1.25)	1.23 (0.97–1.55)	0.138
**Energy-adjusted dietary** **Na intake,** **mg/day**					
Median	509.6	699.1	875.5	1184.2	
Cases/*n*	271/752	247/711	251/704	272/739	
Model 1	1.00	0.89 (0.71–1.11)	0.96 (0.77–1.21)	1.01 (0.81–1.26)	0.772
Model 2	1.00	0.82 (0.65–1.04)	0.90 (0.71–1.13)	0.94 (0.74–1.18)	0.791
Model 3	1.00	0.86 (0.68–1.09)	0.86 (0.68–1.10)	0.88 (0.70–1.12)	0.352
**Energy-adjusted dietary K intake, g/day**					
Median	1.51	1.84	2.12	2.52	
Cases/*n*	261/742	274/728	246/716	260/720	
Model 1	1.00	1.17 (0.94–1.47)	1.03 (0.82–1.29)	1.19 (0.95–1.50)	0.282
Model 2	1.00	1.18 (0.92–1.50)	1.06 (0.82–1.39)	1.32 (0.96–1.80)	0.187
Model 3	1.00	1.17 (0.91–1.50)	1.07 (0.82–1.41)	1.37 (0.99–1.89)	0.123

Model 1: Adjusted for age (year) and sex; Model 2: Further adjusted for education level (≤6, 7–12, and >12 years), monthly income (<1500, 1500–3000, and >3000 yuan), smoking (Y/N), alcohol drinking (Y/N), physical activity(MET), total energy (kcal), fiber (g/day), and saturated fat intake (g/day); Model 3: Further adjusted for waist circumference, total cholesterol, triglycerides, HDL-c, LDL-c, fasting glucose and serum uric acid (all continuous), hypertension status (Y/N) and antihypertensive medication (Y/N); ^a^ Linear trend across increasing quartiles was tested by assuming median values of quartiles as continuous variables.

**Table 3:** Odds ratios and 95% CI for the prevalence of carotid intima-media thickening by quartiles (Q) of sodium (Na) and potassium (K).

	Odds Ratios (95% CI) for Increase in CCA IMT					Odds Ratios (95% CI) for BIF IMT Thickening				
	Q1	Q2	Q3	Q4	*p*-Trend	Q1	Q2	Q3	Q4	*p*-Trend
**Urinary Na/Cr**										
Cases/*n*	260/711	297/729	296/696	330/723		415/711	450/729	426/696	488/723	
Model 1	1.00	1.24 (0.99–1.56)	1.29 (1.03–1.62)	1.46 (1.16–1.83)	0.002	1.00	1.22 (0.98–1.52)	1.17 (0.93–1.46)	1.57 (1.25–1.97)	<0.001
Model 2	1.00	1.23 (0.98–1.55)	1.30 (1.03–1.64)	1.48 (1.18–1.87)	0.001	1.00	1.20 (0.96–1.50)	1.15 (0.92–1.45)	1.55 (1.23–1.96)	0.001
Model 3	1.00	1.20 (0.95–1.52)	1.22 (0.96–1.55)	1.33 (1.05–1.69)	0.026	1.00	1.19 (0.95–1.50)	1.11 (0.88–1.40)	1.43 (1.13–1.81)	0.009
**Urinary K/Cr**										
Cases/*n*	308/732	284/723	296/698	295/706		469/732	438/723	430/698	442/706	
Model 1	1.00	0.96 (0.77–1.20)	1.15 (0.92–1.45)	1.11 (0.88–1.40)	0.195	1.00	0.96 (0.77–1.20)	1.06 (0.84–1.33)	1.12 (0.88–1.41)	0.249
Model 2	1.00	0.97 (0.77–1.21)	1.15 (0.92–1.46)	1.11 (0.88–1.41)	0.203	1.00	0.96 (0.76–1.20)	1.06 (0.84–1.34)	1.10 (0.87–1.40)	0.294
Model 3	1.00	1.01 (0.80–1.28)	1.14 (0.90–1.45)	1.12 (0.88–1.44)	0.245	1.00	0.97 (0.77–1.23)	1.04 (0.82–1.32)	1.11 (0.87–1.41)	0.332
**Urinary Na/K**										
Cases/*n*	256/701	280/712	344/725	303/721		408/701	433/712	466/725	472/721	
Model 1	1.00	1.04 (0.83–1.31)	1.40 (1.12–1.75)	1.13 (0.90–1.41)	0.072	1.00	1.05 (0.84–1.31)	1.15 (0.92–1.44)	1.24 (0.99–1.55)	0.039
Model 2	1.00	1.05 (0.84–1.32)	1.41 (1.13–1.77)	1.15 (0.91–1.44)	0.053	1.00	1.05 (0.84–1.31)	1.14 (0.91–1.42)	1.25 (1.00–1.56)	0.039
Model 3	1.00	1.01 (0.80–1.28)	1.28 (1.01–1.61)	1.03 (0.82–1.31)	0.390	1.00	1.01 (0.81–1.27)	1.06 (0.84–1.33)	1.14 (0.90–1.43)	0.247
**Energy-adjusted dietary** **Na intake,** **mg/day**										
Cases/*n*	306/752	282/711	284/704	333/739		437/752	443/711	440/704	485/739	
Model 1	1.00	0.90 (0.72–1.12)	0.97 (0.78–1.20)	1.19 (0.96–1.47)	0.098	1.00	1.16 (0.93–1.44)	1.21 (0.97–1.50)	1.37 (1.11–1.71)	0.005
Model 2	1.00	0.87 (0.69–1.10)	0.95 (0.76–1.20)	1.13 (0.90–1.42)	0.193	1.00	1.13 (0.90–1.42)	1.17 (0.93–1.47)	1.31 (1.04–1.64)	0.022
Model 3	1.00	0.93 (0.73–1.18)	0.94 (0.74–1.19)	1.10 (0.87–1.39)	0.419	1.00	1.18 (0.93–1.48)	1.15 (0.91–1.45)	1.28 (1.02–1.62)	0.056
**Energy-adjusted dietary K intake, g/day**										
Cases/*n*	317/742	297/728	309/716	282/720		458/742	469/728	446/716	432/720	
Model 1	1.00	0.95 (0.77–1.19)	1.08 (0.87–1.35)	0.96 (0.77–1.20)	0.965	1.00	1.19 (0.95–1.48)	1.10 (0.88–1.37)	1.05 (0.84–1.31)	0.853
Model 2	1.00	0.97 (0.76–1.22)	1.07 (0.83–1.39)	0.98 (0.72–1.33)	0.884	1.00	1.13 (0.89–1.43)	1.03 (0.79–1.33)	1.00 (0.74–1.35)	0.790
Model 3	1.00	0.94 (0.74–1.21)	1.05 (0.81–1.38)	0.98 (0.71–1.34)	0.897	1.00	1.13 (0.89–1.45)	1.04 (0.80–1.36)	1.01 (0.74–1.37)	0.860

Model 1, 2, and 3 refer to Table 2.

**Table 4:** Multivariate-adjusted odds ratios (95% CI) for carotid intima-media thickening by quartiles (Q) of urinary sodium/creatinine ratio stratified by gender, body mass index and hypertension status ^a^.

	*n*	Odds Ratios (95% CI)				*P*_1_^b^	*P*_2_^c^
		Q1	Q2	Q3	Q4		
***Carotid plaque***							
Gender							
Male	904	1.00	0.66 (0.44–0.98)	0.81 (0.54–1.21)	1.02 (0.67–1.56)	0.787	0.484
Female	1907	1.00	1.29 (0.95–1.74)	1.16 (0.86–1.58)	1.38 (1.01–1.89)	0.087	
Body mass index, kg/m^2^							
<25	1965	1.00	0.86 (0.64–1.15)	1.07 (0.80–1.43)	0.99 (0.73–1.33)	0.712	0.074
≥25	850	1.00	1.29 (0.83–2.00)	1.18 (0.75–1.86)	1.87 (1.16–3.01)	0.020	
Hypertension ^d^							
Yes	1086	1.00	0.97 (0.68–1.40)	0.99 (0.69–1.43)	1.52 (1.05–2.20)	0.031	0.132
No	1729	1.00	1.01 (0.73–1.40)	1.22 (0.88–1.68)	1.00 (0.72–1.40)	0.706	
***CCA thickening***							
Gender							
Male	904	1.00	0.79 (0.53–1.20)	1.09 (0.72–1.65)	1.10 (0.70–1.70)	0.434	0.945
Female	1907	1.00	1.05 (0.78–1.41)	1.21 (0.90–1.63)	1.23 (0.90–1.67)	0.129	
Body mass index, kg/m^2^							
<25	1965	1.00	1.58 (1.18–2.12)	1.48 (1.09–1.99)	1.66 (1.21–2.28)	0.005	0.006
≥25	850	1.00	0.72 (0.47–1.11)	0.71 (0.46–1.12)	0.80 (0.50–1.27)	0.366	
Hypertension							
Yes	1086	1.00	1.26 (0.87–1.83)	1.22 (0.84–1.77)	1.46 (1.00–2.13)	0.071	0.897
No	1729	1.00	1.41 (1.03–1.93)	1.15 (0.84–1.58)	1.20 (0.87–1.66)	0.587	
***BIF thickening***							
Gender							
Male	904	1.00	0.69 (0.45–1.06)	1.04 (0.66–1.65)	1.05 (0.64–1.71)	0.523	0.938
Female	1907	1.00	1.05 (0.80–1.37)	1.06 (0.81–1.39)	1.36 (1.03–1.81)	*0.042*	
Body mass index, kg/m^2^							
<25	1965	1.00	1.21 (0.92–1.58)	1.00 (0.76–1.31)	1.37 (1.03–1.82)	0.114	0.548
≥25	850	1.00	1.12 (0.72–1.74)	1.24 (0.78–1.97)	1.38 (0.85–2.24)	0.172	
Hypertension							
Yes	1086	1.00	1.28 (0.86–1.92)	1.10 (0.74–1.63)	1.33 (0.89–1.99)	0.280	0.463
No	1729	1.00	1.21 (0.91–1.60)	1.07 (0.80–1.42)	1.47 (1.10–1.97)	0.032	

^a^ Covariates adjusted for: see Model 3 in Table 2, except for menopause status (Y/N) and passive smoking (Y/N) were further adjusted for in female, but exclude adjusted for smoking and alcohol drinking in female; exclude adjusted for antihypertensive medication in the hypertension-stratified analyses; ^b^ *p*-values for trend; ^c^ *p*-values for interaction; ^d^ Hypertension was defined as SBP ≥ 140 mmHg or DBP ≥ 90 mmHg or use of an antihypertension drug.

## Supplementary Materials

The following are available online at http://www.mdpi.com/2072-6643/8/10/612/s1, Supplementary Materials Tables S1–S3: Table S1: Characteristics of participants by quartiles (Q) of urinary potassium/creatinine ratio; Table S2: Odds ratios and 95% CI for the prevalence of increased carotid IMT by quartiles (Q) of sodium (Na) without those using antihypertension medication; Table S3: Odds ratios and 95% CI for the prevalence of increased carotid IMT by quartiles (Q) of potassium (K) without those using antihypertension medication.

## Abbreviations

The following abbreviations are used in this manuscript:

95% CI	95% confidence interval
CCA	common carotid artery
CHD	coronary heart disease
Cr	creatinine
CVD	cardiovascular disease
FFQ	food frequency questionnaire
HDL-c	high-density lipoprotein cholesterol
IMT	intima-media thickness
K	potassium
LDL-c	low-density lipoprotein cholesterol
Na	sodium
