# Supplementary material for: Urinary Sodium and Potassium Excretion and Carotid Atherosclerosis in Chinese Men and Women
Source: Nutrients. 2016 Oct 1;8(10):612. doi: 10.3390/nu8100612 (PMC5084000; doi:10.3390/nu8100612)
Supplement: Supplementary file 1 [file nutrients-08-00612-s001.docx]

Supplementary Materials: Urinary Sodium and Potassium Excretion and Carotid Atherosclerosis in Chinese Men and Women

Xiao-Wei Dai, Cheng Wang, Ying Xu, Ke Guan, Yi-Xiang Su and Yu-Ming Chen

**Table S1.** Characteristics of participants by quartiles (Q) of urinary potassium/creatinine ratio (*n* = 3290).

|  | **Q1  Mean ± SD /*n* (%)** | **Q2  Mean ± SD /*n* (%)** | **Q3  Mean ± SD /*n* (%)** | **Q4  Mean ± SD /*n* (%)** | ***p*-Trend** |
| --- | --- | --- | --- | --- | --- |
| Urinary K/Cr | 2.1 ± 0.4 | 3.1 ± 0.2 | 4.1 ± 0.3 | 6.8 ± 4.8 | <0.001 |
| Age, year | 59.6 ± 5.8 | 60.2 ± 6.0 | 60.3 ± 6.0 | 61.1 ± 6.1 | <0.001 |
| Male, *n* (%) | 451 (54.9) | 294 (35.7) | 187 (22.7) | 135 (16.4) | 0.108 |
| Waist circumference, cm | 84.6 ± 9.1 | 84.9 ± 8.7 | 85.2 ± 8.6 | 85.2 ± 8.8 | 0.337 |
| Body mass index, kg/m^2^ | 23.6 ± 3.2 | 23.5 ± 3.0 | 23.7 ± 3.2 | 23.7 ± 3.2 | 0.526 |
| Overweight, *n* (%) | 248 (30.2) | 231 (28.1) | 258 (31.3) | 249 (30.3) | 0.692 |
| Education >2 years, *n* (%) | 211 (25.7) | 209 (25.4) | 208 (25.3) | 209 (25.4) | 0.046 |
| **Monthly income, *n* (%)** | | | | | |
| <1500, yuan | 61 (7.4) | 72 (8.7) | 41 (5.0) | 50 (6.1) |  |
| 1500–3000, yuan | 416 (50.6) | 385 (46.8) | 401 (48.7) | 397 (48.3) |  |
| >3000, yuan | 344 (41.8) | 365 (44.3) | 379 (46.1) | 373 (45.4) |  |
| Current smoker, *n* (%) | 162 (19.7) | 82 (10.0) | 46 (5.6) | 28 (3.4) | <0.001 |
| Alcohol users, *n* (%) | 95 (11.6) | 64 (7.8) | 59 (7.2) | 40 (4.9) | <0.001 |
| Physical activity, MET ^c^ | 25.0 ± 6.5 | 25.2 ± 7.1 | 25.0 ± 6.8 | 24.8 ± 6.4 | 0.457 |
| Urinary Na/Cr | 12.3 ± 6.3 | 15.4 ± 7.2 | 18.2 ± 9.0 | 25.1 ± 19.2 | <0.001 |
| Urinary Cr, mmol/L | 9.5 ± 5.0 | 7.1 ± 3.7 | 5.8 ± 3.2 | 4.5 ± 2.6 | <0.001 |
| Urinary Na/K | 5.9 ± 3.0 | 4.9 ± 2.3 | 4.5 ± 2.2 | 3.8 ± 2.0 | <0.001 |
| SBP, mm Hg | 125.2 ± 18.0 | 125.3 ± 17.6 | 125.1 ± 18.5 | 125.2 ± 17.7 | 0.990 |
| DBP, mm Hg | 76.2 ± 10.6 | 75.7 ± 10.4 | 75.4 ± 10.2 | 74.6 ± 10.0 | 0.001 |
| TC, mmol/L | 5.47 ± 1.01 | 5.49 ± 1.00 | 5.66 ± 1.05 | 5.66 ± 1.11 | <0.001 |
| TG, mmol/L | 1.54 ± 1.25 | 1.55 ± 1.40 | 1.57 ± 1.29 | 1.49 ± 0.97 | 0.455 |
| HDLc, mmol/L | 1.38 ± 0.40 | 1.43 ± 0.40 | 1.44 ± 0.41 | 1.50 ± 0.41 | <0.001 |
| LDLc, mmol/L | 3.53 ± 0.90 | 3.51 ± 0.88 | 3.62 ± 0.89 | 3.62 ± 0.94 | 0.012 |
| Fasting glucose, mmol/L | 5.03 ± 1.28 | 5.00 ± 1.18 | 5.03 ± 1.17 | 5.08 ± 1.23 | 0.307 |
| **Carotid IMT** | | | | | |
| CCA, mm | 0.920 ± 0.113 | 0.918 ± 0.118 | 0.930 ± 0.118 | 0.922 ± 0.124 | 0.418 |
| BIF, mm | 0.958 ± 0.125 | 0.952 ± 0.118 | 0.957 ± 0.123 | 0.968 ± 0.139 | 0.149 |
| Plaque, *n* (%) | 290 (35.3) | 226 (27.5) | 252 (30.6) | 263 (32.0) | 0.009 |

Abbreviations: Refer to Table 1.

**Table S2.** Odds ratios and 95% CI for the prevalence of increased carotid IMT by quartiles (Q) of sodium (Na) without those using antihypertension medication.

|  | **Odds Ratios (95% CI) by Quartiles of Urinary Na/Cr** | | | | |
| --- | --- | --- | --- | --- | --- |
|  | **Q1** | **Q2** | **Q3** | **Q4** | ***p*-Trend ^a^** |
| **Median** | **7.9** | **12.7** | **17.8** | **26.8** |  |
| **Carotid plaque** | | | | | |
| Cases/*n* | 155/528 | 167/542 | 172/528 | 169/541 |  |
| Model 1 | 1.00 | 1.09 (0.82–1.43) | 1.22 (0.92–1.61) | 1.10 (0.83–1.46) | 0.377 |
| Model 2 | 1.00 | 1.11 (0.84–1.47) | 1.27 (0.96–1.68) | 1.13 (0.85–1.50) | 0.287 |
| Model 3 | 1.00 | 1.10 (0.83–1.47) | 1.25 (0.94–1.66) | 1.10 (0.82–1.47) | 0.391 |
| **Increase in CCA IMT** | | | | | |
| Cases/*n* | 170/528 | 206/542 | 197/528 | 213/541 |  |
| Model 1 | 1.00 | 1.32 (1.01–1.73) | 1.31 (1.00–1.72) | 1.40 (1.06–1.83) | **0.027** |
| Model 2 | 1.00 | 1.30 (0.99–1.70) | 1.29 (0.98–1.69) | 1.40 (1.06–1.84) | **0.027** |
| Model 3 | 1.00 | 1.27 (0.96–1.67) | 1.19 (0.90–1.58) | 1.28 (0.97–1.71) | 0.147 |
| **Increase in BIF IMT** | | | | | |
| Cases/*n* | 287/528 | 322/542 | 306/528 | 336/541 |  |
| Model 1 | 1.00 | 1.30 (1.01–1.68) | 1.25 (0.97–1.62) | 1.51 (1.16–1.95) | **0.005** |
| Model 2 | 1.00 | 1.30 (1.00–1.68) | 1.24 (0.96–1.61) | 1.50 (1.15–1.95) | **0.006** |
| Model 3 | 1.00 | 1.27 (0.98–1.65) | 1.17 (0.90–1.52) | 1.38 (1.06–1.81) | **0.043** |

Model 1: Adjusted for age (year) and sex; Model 2: Further adjusted for education level (≤6, 7–12, and >12 years), monthly income (<1500, 1500–3000, and >3000 yuan), smoking(Y/N), alcohol drinking(Y/N), physical activity(MET), total energy (kcal), fiber (g/day), and saturated fat intake (g/day); Model 3: Further adjusted for waist circumference, total cholesterol, triglycerides, HDL-c, LDL-c, fasting glucose, serum uric acid (all continuous), and hypertension status (yes or no); ^a^ Linear trend across increasing quartiles was tested by assuming median values of quartiles as continuous variables.

**Table S3.** Odds ratios and 95% CI for the prevalence of increased carotid IMT by quartiles (Q) of potassium (K) without those using antihypertension medication.

|  | **Odds Ratios (95% CI) by Quartiles of Urinary K/Cr** | | | | |
| --- | --- | --- | --- | --- | --- |
|  | **Q1** | **Q2** | **Q3** | **Q4** | ***p*-Trend ^a^** |
| **Median** | **2.2** | **3.1** | **4.0** | **5.9** |  |
| **Carotid plaque** | | | | | |
| Cases/*n* | 186/542 | 142/537 | 157/524 | 178/536 |  |
| Model 1 | 1.00 | 0.78 (0.59–1.02) | 0.93 (0.70–1.23) | 1.11 (0.84–1.47) | 0.270 |
| Model 2 | 1.00 | 0.77 (0.58–1.01) | 0.93 (0.70–1.23) | 1.10 (0.83–1.47) | 0.286 |
| Model 3 | 1.00 | 0.78 (0.59–1.04) | 0.95 (0.71–1.26) | 1.12 (0.84–1.50) | 0.244 |
| **Increase in CCA IMT** | | | | | |
| Cases/*n* | 201/542 | 184/537 | 203/524 | 198/536 |  |
| Model 1 | 1.00 | 1.01 (0.77–1.32) | 1.23 (0.94–1.62) | 1.14 (0.87–1.51) | 0.184 |
| Model 2 | 1.00 | 1.04 (0.80–1.36) | 1.23 (0.93–1.62) | 1.15 (0.87–1.52) | 0.198 |
| Model 3 | 1.00 | 1.09 (0.82–1.43) | 1.25 (0.94–1.66) | 1.14 (0.85–1.52) | 0.276 |
| **Increase in BIF IMT** | | | | | |
| Cases/*n* | 327/542 | 305/537 | 301/524 | 318/536 |  |
| Model 1 | 1.00 | 1.01 (0.78–1.30) | 1.06 (0.81–1.37) | 1.17 (0.90–1.52) | 0.227 |
| Model 2 | 1.00 | 1.01 (0.78–1.31) | 1.06 (0.81–1.38) | 1.16 (0.89–1.52) | 0.245 |
| Model 3 | 1.00 | 1.02 (0.78–1.33) | 1.06 (0.81–1.39) | 1.14 (0.87–1.50) | 0.332 |

Model 1, 2 and 3 see supplementary materials Table S2.
